# The Challenges of Identifying Fibromyalgia in Adolescents

**DOI:** 10.1155/2022/8717818

**Published:** 2022-04-08

**Authors:** Elisha E. Peterson, Caylynn Yao, Sangeeta D. Sule, Julia C. Finkel

**Affiliations:** ^1^Division of Anesthesiology, Pain and Perioperative Medicine, Children's National Hospital, Washington, DC, USA; ^2^School of Medicine and Health Sciences, George Washington University, Washington, DC, USA; ^3^Division of Rheumatology, Children's National Hospital, Washington, DC, USA; ^4^The Sheikh Zayed Institute for Pediatric Surgical Innovation, Children's National Hospital, Washington, DC, USA

## Abstract

**Aim:**

Fibromyalgia (FM) is a noninflammatory disorder of the nervous system characterized by widespread musculoskeletal pain and somatic complaints of at least 3 months duration. There are no current diagnostic criteria for fibromyalgia in children to guide clinicians in recognition, thus leading to many subspecialty referrals and extensive imaging and tests. The purpose of this retrospective review is to compare two diagnostic criteria for juvenile fibromyalgia.

**Methods:**

A retrospective chart review of 20 children diagnosed with juvenile fibromyalgia from a singular pain physician practice was performed. Both the Yunus diagnostic criteria and the 2016 American College of Rheumatology (ACR) diagnostic criteria were applied and compared.

**Results:**

85% of patients met criteria for fibromyalgia under both criteria. 15% of patients met only ACR criteria as the Yunus criteria excluded those with underlying conditions. Of the children who fulfilled criteria with use of both diagnostic tools, this cohort reported a high somatic symptom burden as demonstrated by the ACR symptom severity scales of 12 and satisfaction of at least 4 Yunus and Masi minor criteria on average. Widespread pain was noted with an ACR Widespread Pain Index (WPI) of 7, and tender points were 4.8 on average across the cohort. Effective therapeutic regimens among patients varied widely from medical monotherapy to multimodal treatment. Patients presented with pain for 1.8 yrs on average prior to a diagnosis. All of the cohort had a normal laboratory evaluation; half the cohort received additional imaging and testing.

**Conclusion:**

This case series suggests the need for an updated diagnostic tool for pediatric fibromyalgia to facilitate recognition and treatment.

## 1. Introduction

The term *fibromyalgia* was developed by Smythe and Moldofsky in the 1970s to reflect a chronic condition where there is no accompanying inflammation but pain involving connective tissues. In the United States, the prevalence of pediatric fibromyalgia steadily increases across the age spectrum from 0.5–1% in those less than 4 years old to up to 6% in the 15–19-year old age group compared to 2% in the adult population [1]. The pain of FM typically presents diffuse or multifocal, difficult to localize, often waxes and wanes, and migratory in nature. Most patients diagnosed with FM also display augmented sensory processing to normally painful and nonpainful stimuli [2]. In a cohort of 148 children, the most common complaints reported include generalized pain, headache, and sleep disturbance [3] in line with observations made by Yunus and Masi in 1985.

FM is a noninflammatory disorder of the nervous system characterized by widespread musculoskeletal pain and somatic complaints of at least 3 months duration [4]; juvenile-onset fibromyalgia carries a greater burden of psychological and functional impairment when compared to adult-onset fibromyalgia [5]. The rate of home schooling is six times the national average in juvenile fibromyalgia, and those who continue to attend school miss up to a month of instruction due to frequent absences [6]. Despite this greater impairment, there is no gold standard for establishing the diagnosis of FM in children [1]. In 1985, Yunus and Masi proposed clinical criteria for juvenile fibromyalgia which included generalized musculoskeletal ache for at least 3 months and psychosomatic symptoms (Table 1) [7]. Although their criteria were not validated [1], similar features are present in the American College of Rheumatology (ACR) diagnostic criteria for FM in adults (Table 1) [4]. The ACR first established standardized criteria for the diagnosis of FM in 1990. It was revised in 2010/2011/2016, no longer requiring discrete tender points on physical exam. Instead, cardinal symptoms of FM now comprise widespread pain, fatigue, sleep disorders, and memory disturbances [4]. The ACR 2016 provisional diagnostic criteria for FM in adults includes a Widespread Pain Index (WPI) and a Symptom Severity scale (SSS). The sum of the WPI and SSS results in a value for the Fibromyalgia Severity (FS) scale where the minimum number must be 12 to satisfy FM criteria [1]. The FS can be tracked over time to identify a response to treatment [1]. Although the ACR criteria are adult-based, they have been used for in pediatric studies due to the lack of an updated and validated criterion for children [8].

It is our aim to contribute case-based evidence of pediatric FM to inform further tailored research into diagnostic criteria and treatment. It is necessary to establish a timely diagnosis for FM given its devastating effects on quality of life [9, 10]. Children with juvenile fibromyalgia miss more school than those with organic diseases [9]. Earlier detection leads to treatment and less impairment, thus improving quality of life. Outcomes are favorable with 73% of pediatric FM patients evaluated after a 30-month follow-up reporting remission [11]. Clarity regarding the most appropriate pediatric criteria to promote identification is paramount.

## 2. Case Presentation

A retrospective chart review of 20 patients with a new diagnosis of FM between 2018 and 2019 from a single pediatric pain medicine physician at Children's National Hospital is described. The Yunus diagnostic criteria and the 2016 ACR criteria for juvenile fibromyalgia syndrome [4] were applied (Table 1). Patients 18-year-old or less with a diagnosis of juvenile fibromyalgia were included for review. The average age was 14 years old (Table 2), the onset of puberty. The cohort was overwhelmingly female with half of the patients identified as Caucasian and the other half as people of color (Table 2). The duration of pain prior to presentation had a mean duration of nearly 2 years. During which time, all the patients received at least one diagnostic test (Table 2). The majority of the cohort were absent from school signifying functional impairment. Over half of patients had a family history of chronic pain, and a third had family strain or divorce ongoing at the time of evaluation (Table 2). The average treatment duration was 8 months (Table 2). The types of therapy offered were medications, physical therapy, cognitive therapy, injections, and acupuncture. On average, two modes of therapy were required in the treatment of FM (Table 2).

## 3. Discussion

The ACR criteria are frequently used in pediatric clinical practice and literature despite being validated for the adult population only, while the Yunus criteria are the only criterion developed from the evaluation of children. We found that use of both ACR and Yunus criteria has limitations in the recognition of FM in children. The limitations found with the Yunus criteria include exclusion for underlying conditions. Patients with cooccurring autoimmune disorders were included in this cohort because, despite their diagnostic evaluations demonstrating stable autoimmune disease, they had severe pain and disability that was captured using ACR criteria. The Yunus criteria have not been updated since their development in 1985, whereas the ACR criteria have underwent multiple revisions but were not developed for use in children.

The limitations found within both criteria is the minimal duration of pain for 3 months. Chronic pain is a dynamic neuroplastic process where the imbalance in nociceptive processing favors a pronociceptive profile [12]. Given that neuroplasticity is maximal at 2 years of age and synaptic pruning is not complete until 16 years of age [13], the evidence of maladaptive plasticity can be seen earlier than 12 weeks. FM is a chronic neuropathic pain condition, thus also dynamic in nature. Adhering to the rigidity of a time cut off is ill fitting for children. Due to the high burden of somatic symptoms, functional impairment, and pain for 11 weeks, the one male patient in our cohort was included in the review and was diagnosed with FM and improved with treatment.

In the adult population, the prevalence of FM is 1.5 to 2 times as often in women than in men, frequently making its first appearance during menopause [2]. Consistent with the adult literature, an overwhelming proportion in our cohort are female. Unlike adults where symptoms manifest for women around menopause, it is appreciated that menstruation was a hormonal trigger that flared pain and/or the initial trigger within this cohort of patients. The mean age of diagnosis in this cohort is 14.8 years. This is in line with current literature where the mean age of FM diagnosis in adolescents is between 13.7 and 15.5 years [14]. Dysmenorrhea was an overlooked symptom in almost a third of the 18 females in the cohort which responded to oral contraceptives. In 3 patients, CBT and OCPs were effective treatments for their FM.

The unique parental aspect has further been considered in pediatric FM, in which youths with FM come from families described as anxious, disorganized, or with a high degree of parental control, exerting an influence on daily coping mechanisms [15, 16]. Family environmental factors are thus unique and salient to understanding pediatric FM. 60% of children in this cohort endured parental divorce or a single household where parental strain is reported. It highlights the need for greater family support within the medical system. The data suggest children should not be viewed in isolation but instead as a microcosm of the collective family unit.

The management of pediatric FM currently centers on nonpharmacologic measures such as behavioral and physical rehabilitation. In a cohort of 64 children 13 to 18 years of age who were diagnosed with fibromyalgia using ACR adult criteria, over half of the children had improved pain after completion of a 10–36 day intensive physical and psychosocial therapy program and retained improved function with no interventions or medications provided–the study concluded that interventions or medications result in overmedicalization of these patients [8]. However, access to pain rehabilitation programs is a real barrier as this study reported a 97 to 233 day wait prior to program entry [8]. Many patients presented to our clinic with pain that interfered with their ability to participate in physical therapy. As such, medication and intervention as appropriate were offered to facilitate participation in therapy.

Effective regimens for fibromyalgia included a medication in our cohort. Duloxetine, milnacipran, and pregabalin are FDA approved for fibromyalgia with duloxetine for use in children as young as 7 years of age. Use of pregabalin for the treatment of fibromyalgia in pediatrics has not been established, but a placebo-controlled trial in adolescents as young as 12 years old demonstrated pregabalin to be as safe as its use in adults [17]. Duloxetine was either not effective or not tolerated in 23% of patients; they were switched to a tricyclic antidepressant, pregabalin, or milnacipran with better results. Although both duloxetine and milnacipran are both SNRIs, they differ in their reuptake inhibition as duloxetine is more selective for serotonin reuptake and milnacipran for norepinephrine reuptake [18]. SNRIs, SSRIs, and neuropathic pain agents have a black box warning for increased risk of suicidal thoughts; families should be made aware of this warning as patients with FM have a high association of comorbid depression. Low dose naltrexone has been found to be a safe and effective medication in decreasing symptom severity in adults [19]. It acts on the microglia in low doses and is well tolerated. There are additional implications in immune dysfunction in decreasing autoimmunity through its actions on B and T cells [20]. 15% of patients in this cohort received benefit from it. Low dose naltrexone was compounded into 1.5 mg capsules and instructed to be increased by 1 capsule every 2 weeks if there is no benefit, upto 3 capsules maximum.

Myofascial pain, FM, and hypermobility syndromes can coexist in patients who present with soft tissue pain [21]. Hypermobility syndrome is a well-known clinical association of pediatric FM as 81% of children diagnosed with FM met criteria for hypermobility in a study of 338 children [22]; a quarter of our cohort has hypermobility. Loose joints lead to tight muscles; trigger point mediated pain was noted in our cohort. Trigger points are taut bands present within muscles that reproduce pain on palpation and can radiate pain in a nondermatomal fashion [21]. One patient in our cohort demonstrated a trigger point which led to a presumed failure of physical therapy. The use of trigger point injections allowed greater engagement in therapies.

Our small sample size is a limitation of this study. Demographically, our population differs from previous studies that report over 90% Caucasian representation within juvenile FM [7, 8], whereas in our cohort it was 50%. Such a difference could be due to the sample size or, given the location of our hospital, a greater diversity of patients is captured. Another limitation is the lack of distinction between primary fibromyalgia and secondary fibromyalgia which undoubtedly adds additional heterogeneity to an already multifaceted condition. FM is a clinical condition to minimize variances in documentation within history taking and physical examination, review of medical records from one pain physician as reviewed. However, the retrospective nature of this review with a review of records from one physician can lead to bias. Larger prospective studies of FM in children can provide insights into FM that could lead to the development of pediatric-specific FM criteria.

This case series demonstrates the need for personalized treatment within the management of FM in children. Although use of a rehabilitative model is encouraged in all children with chronic pain, high pain severity can limit participation in these therapies. For patients who were unable to participate in therapy due to high pain burden, the use of pharmacologic and interventional supports facilitated our patients' engagement in therapy. Dysmenorrhea was an often overlooked driver for FM, the treatment of which decreased functional impairments. This case series also highlights the extent of diagnostic testing and specialty evaluations which delayed treatment by over 1.5 years in this cohort. Many children with widespread musculoskeletal pain are referred to a pediatric rheumatologist for evaluation for juvenile idiopathic arthritis (JIA), but in the absence of other systemic signs, pain has a high negative predictive value for an autoimmune etiology; joint swelling is more predictive [23]. By refining our diagnostic criteria, invasive and lengthy diagnostic evaluations of patients with chronic pain can be minimized. Due to the wide range of symptomatology and subjectivity, there is a clear need for uniform, consistent diagnostics to capture the heterogeneity of expression in FM so the pathology is not disputed. Major gaps exist in diagnostics for pediatric FM as children carry entirely different social, biological, physical, behavioral, developmental, and emotional biospheres than adults. Improved diagnostics would hasten identification, decreasing the time to treatment and thus improving quality of life.

## Figures and Tables

**Table 1:** Comparison of diagnostic criteria for FM.

Author	Major criteria	Minor criteria	Clinical diagnosis
Yunus and Masi [7]	(1) Generalized musculoskeletal aching at 3 or more sites (2) Duration of 3 months or more (3) Normal labs (4) No underlying condition	(1) Chronic anxiety (2) Fatigue (3) Poor sleep (4) Chronic headaches (5) Irritable bowel syndrome (6) Subjective soft tissue swelling (7) Numbness (8) Pain modulation by: (i) Physical activity (ii) Anxiety/stress (iii) Weather	Juvenile FM: 4 major with 5 tender points +3 minor Or 4 tender points + 5 minor
Wolfe et al. [4]	N/A	N/A	FM: (1) Generalized pain in at least 4 of 5 regions (2) Symptoms present for at least 3 months (3) WPI >/ = 7 and SSS >/ = 5 or WPI 4–6 and SSS >/ = 9 (4) FM diagnosis is valid irrespective of other diagnoses

**Table 2:** Demographic and clinical characteristics (*n* = 20).

Age category, *y*, *n* (%)	
10–14	8 (40%)
15–19	12 (60%)
Sex, *n* (%)	
Male	1 (5%)
Female	19 (95%)
Race, *n* (%)	
White	10 (50%)
Black	4 (20%)
Hispanic	3 (15%)
Asian	3 (15%)
Pain duration, mo	22 (16)
Pain in family history, *n* (%)	12 (60%)
Parental divorce or strain, *n* (%)	7 (35%)
Number of diagnostic tests	1.6 (0.6)
Full school attendance unchanged academic performance, *n* (%)	5 (25%)
WPI	7 (1.4)
SSS	12 (2.8)
Yunus criteria met, *n* (%)	17 (85%)
Treatment modalities	2.4 (0.99)
Functional restoration, mo	8.6 (3.4)

The data are presented as the mean (standard deviation) or *n* (%), as indicated. Type of testing each count as 1: labs, imaging, and dynamic testing. WPI: Widespread Pain Index; SSS: Symptom Severity Scale. Treatment modalities: medications^*∗*^physical therapy, cognitive therapy, injections, and acupuncture. ^*∗*^Duloxetine, pregabalin, gabapentin, milnacipran, low dose naltrexone, and birth control pill.

## Data Availability

The data are available from the corresponding author on reasonable request.

## Abbreviation

FS: Fibromyalgia Severity Scale
CAM: Complementary alternative modalities
PANS: Pediatric acute onset neuropsychiatric syndrome
CBT: Cognitive behavioral therapy
PT: Physical therapy
SNRI: Serotonin norepinephrine reuptake inhibitor
SSRI: Selective serotonin reuptake inhibitor
TCA: Tricyclic antidepressant
OCP: Oral contraceptive pills
FM: Fibromyalgia
POTS: Postural orthostatic tachycardia syndrome
EDS: Ehlers-Danlos syndrome
OI: Orthostatic intolerance
JIA: Juvenile idiopathic arthritis
IBS: Irritable bowel syndrome
WPI: Widespread Pain Index
ACR: American College of Rheumatology
SSS: Symptom Severity Score.
